# Plant-food components at the microbiota–neuroinflammation interface: human evidence, translational limits, and sustainable dietary translation

**DOI:** 10.3389/fnut.2026.1907913

**Published:** 2026-09-01

**Authors:** Kingsley Excel Dunga, Sylvester Chibueze Izah, Afamefuna Dunkwu-Okafor, Vivian Ibienebakabobo Promise, Esther Ugo Alum, Olugbemiga Ojo Aliu, Ebinyo Rebecca Aseibai, Matthew Chidozie Ogwu

**Affiliations:** 1 Department of Medical Laboratory Science, Kingsley Ozumba Mbadiwe University, Ogboko, Ideato, Imo, Nigeria; 2 Department of Community Medicine, Faculty of Clinical Sciences, Bayelsa Medical University, Yenagoa, Bayelsa, Nigeria; 3 Department of Microbiology, Faculty of Life Sciences, University of Benin, Benin, Edo, Nigeria; 4 Department of Public Health, Faculty of Health Sciences, Bayelsa Medical University, Yenagoa, Bayelsa, Nigeria; 5 Department of Research and Publications, Kampala International University, Kampala, Uganda; 6 Department of Environmental Biology, Auchi Polytechnic, Auchi, Edo, Nigeria; 7 Department of Microbiology, Federal University Otuoke, Otuoke, Bayelsa, Nigeria; 8 Goodnight Family Department of Sustainable Development, Appalachian State University, Boone, NC, United States

**Keywords:** bioavailability, microbiota-gut-brain axis, neuroinflammation, neurological nutrition, NLRP3 inflammasome, plant foods, short-chain fatty acids, sustainable diets

## Abstract

This narrative review evaluates how convincingly plant-food components and plant-rich dietary patterns influence neuroinflammation and whether those mechanisms translate into neurological benefit. Infection is treated as one possible initiating exposure, neuroinflammation as a context-dependent mechanism, and neurological complications as clinical outcomes. Targeted literature searches and reference verification prioritized original human trials, mechanistic studies and balanced evidence syntheses. The most coherent pathways are modulation of microglial inflammatory signaling, including NLRP3-related processes, by selected phytochemicals, and microbial production of short-chain fatty acids from fermentable fiber, with effects on intestinal barrier function, systemic immune tone and microglial homeostasis. Cell and animal evidence is substantial, but human findings are heterogeneous. Randomized studies of whole dietary patterns and selected foods report both favorable and null cognitive, vascular, inflammatory, and microbiome outcomes. Trials of formulated curcumin and resveratrol show selective signals rather than consistent disease-modifying efficacy, underscoring problems of dose, metabolism, formulation and target engagement. Direct human evidence that plant foods prevent or modify infection-specific neurological outcomes remains insufficient. Translation also depends on food-system conditions: affordability, cultural acceptability, local availability, processing, safety, environmental burden and the foods displaced by a plant-forward intervention. Plant foods should therefore be framed as components of nutritionally adequate and context-specific sustainable diets, not as universally low-impact products or substitutes for clinical care. Future studies should integrate defined food exposures, pharmacokinetics, microbial function, inflammatory and vascular measures, neurological outcomes, safety, cost and environmental indicators.

## 1 Introduction

Diet and brain health are related through a broad network of metabolic, vascular, immune and microbial pathways. However, the proposition that plant foods are generally beneficial for the brain is no longer a sufficiently precise review question. Fruits, vegetables, legumes, whole grains, nuts, seeds, herbs, spices and edible fungi differ greatly in composition, and their effects cannot be inferred from the antioxidant capacity of an extract or from the presence of a single phytochemical. A useful synthesis must distinguish exposure, mechanism, evidence level and outcome. It must also separate the effects of whole dietary patterns from those of purified compounds, because food structure, dose, repeated intake, metabolism and substitution effects influence biological response (1–3).

This review therefore asks whether functional plant foods can modulate neuroinflammation through defined pathways and whether those pathways provide credible support for neurological resilience. Two mechanisms receive particular emphasis. First, several polyphenols and related phytochemicals influence nuclear factor kappa B, mitogen-activated protein kinase and NLRP3 inflammasome signaling in experimental systems. These pathways affect the production of interleukin-1 beta, interleukin-6, tumor necrosis factor-alpha and other mediators involved in microglial and astrocytic responses (4–6). Second, fermentable dietary fiber can increase microbial short-chain fatty acids, especially acetate, propionate and butyrate, which influence epithelial integrity, immune regulation and microglial maturation (7–9).

These pathways are biologically coherent, but biological coherence is not the same as demonstrated clinical efficacy. Cell cultures frequently use concentrations that are difficult to achieve through ordinary diets. Animal models simplify complex human exposures and may not reproduce comorbidity, polypharmacy or long-term dietary behavior. Observational studies are vulnerable to residual confounding because high adherence to plant-rich diets often clusters with education, income, physical activity, non-smoking, preventive healthcare and other favorable behaviors. Randomized dietary trials improve causal inference, but they differ in duration, baseline risk, adherence, comparator diets and outcome selection. Consequently, evidence should be graded rather than presented as a continuous body of equally persuasive findings.

The role of infection requires similar discipline. Bacterial, viral and parasitic infections can initiate central or peripheral inflammatory responses, and excessive or persistent neuroinflammation can contribute to encephalopathy, cognitive impairment, mood changes, seizures or other neurological complications. Yet evidence that a food or dietary pattern prevents an infection-specific neurological endpoint is not established merely because a phytochemical suppresses lipopolysaccharide-induced cytokines in microglia. Lipopolysaccharide challenge is an inflammatory model, not a complete model of live infection, pathogen clearance, antimicrobial treatment or clinical recovery. The infection-related part of this review is therefore presented as a translational application of the general neuroinflammatory framework, with the limits of extrapolation stated explicitly (10, 11).

The term functional plant food is used here for edible plant-derived foods or ingredients that provide nutrients and bioactive constituents capable of influencing physiological processes beyond the prevention of overt deficiency. Edible mushrooms are fungi and are discussed separately as plant-associated foods. The term functional does not imply proven treatment. Neuroinflammation denotes coordinated immune and glial responses within the central nervous system; infection is an aetiological trigger; and an infection-related neurological complication is an outcome that may arise through direct invasion, vascular injury, systemic inflammation, immune dysregulation or their interaction. The word disease is reserved for established diagnoses, such as Alzheimer's disease or Parkinson's disease, while disorder or complication is used for broader clinical categories.

### 1.1 Review approach and evidence appraisal

This article is a critical narrative review rather than a systematic review or meta-analysis. Targeted searches of PubMed, publisher databases and cited-reference chains were conducted through July 2026. Search combinations included plant food, functional food, polyphenol, flavonoid, curcumin, resveratrol, dietary fiber, short-chain fatty acid, microbiota-gut-brain axis, microglia, NLRP3, neuroinflammation, cognition, infection, sustainable diet, affordability and food system. Peer-reviewed original human trials were prioritized for clinical claims; original mechanistic studies were used to define pathways; and recent reviews were retained when they provided broad synthesis. Reference titles, author details, journal information and DOI records were checked against publisher or PubMed records.

Evidence was appraised hierarchically. Clinically meaningful neurological outcomes in adequately controlled human studies were given greater weight than peripheral biomarkers, observational associations, animal outcomes, cell-culture responses or computational predictions. Claims such as prevents, reduces or improves are used only when the cited study directly measured the stated outcome; otherwise, the wording is limited to association, mechanistic support or biological plausibility. This approach permits inclusion of heterogeneous evidence while preventing experimental potency, food composition or network-pharmacology predictions from being presented as established human efficacy.

## 2 Cause, mechanism, and outcome in neuroinflammatory biology

### 2.1 Neuroinflammation as a context-dependent response

Neuroinflammation is neither uniformly harmful nor synonymous with neurological disease. Microglia and astrocytes participate in immune surveillance, removal of cellular debris, synaptic remodeling, metabolic support and repair. During acute infection or injury, pattern-recognition receptors detect pathogen-associated or damage-associated signals and initiate responses intended to contain the threat. Problems arise when activation is excessive, poorly resolved or repeatedly amplified. Persistent cytokine production, reactive oxygen and nitrogen species, complement activation, altered glutamate handling and mitochondrial stress can impair synapses and increase neuronal vulnerability. This dynamic interpretation is more accurate than a simple division between protective and destructive glial states (6, 12).

Peripheral inflammation can communicate with the brain through circulating cytokines, endothelial signaling, vagal pathways, circumventricular organs and changes in blood-brain barrier function. A systematic review of animal experiments found that peripheral inflammatory challenges robustly induce microglial activation, although the pattern depends on the stimulus, dose and time point (10). This observation supports the plausibility of diet-mediated modulation of systemic inflammatory tone, but it does not demonstrate that a dietary intervention will prevent a neurological complication during a specific infection. The initiating stimulus and the host response must be studied together.

Microglial activation is often described using a binary pro-inflammatory and anti-inflammatory vocabulary. Current evidence indicates a more heterogeneous spectrum of transcriptional and functional states. For nutritional research, the relevant question is therefore not whether a compound switches microglia from one fixed phenotype to another, but whether it alters inflammatory thresholds, metabolic capacity, phagocytosis, cytokine output or interactions with astrocytes and endothelium. This distinction is important because some immune activity is necessary for pathogen defense, while broad suppression could be counterproductive during active infection (6, 12).

### 2.2 Blood-brain barrier and neurovascular interfaces

The blood-brain barrier is formed by endothelial cells, pericytes, astrocytic end-feet and basement-membrane components that regulate exchange between blood and neural tissue. Cytokines, oxidative stress, microbial products and endothelial injury can alter tight-junction proteins and transport functions, increasing exposure of the central nervous system to peripheral inflammatory signals. Infection and systemic inflammation therefore affect the brain even when a pathogen does not directly invade neural tissue (11). Experimental work in germ-free mice also indicates that the intestinal microbiota contributes to blood-brain barrier development and permeability, although translation from germ-free models to human dietary intervention must remain cautious (13). Braniste et al. (13) found greater blood-brain barrier permeability and lower occludin and claudin-5 expression in germ-free mice than in conventionally colonized mice; recolonization reduced permeability and increased tight-junction protein expression. These findings establish microbiota-dependent barrier regulation in mice, but they do not quantify the effect of a plant-food intervention in humans.

Neurovascular coupling links neuronal activity with local blood flow and substrate delivery. Endothelial dysfunction, impaired nitric oxide signaling and vascular inflammation can reduce this adaptive response. Plant-rich dietary patterns may influence these pathways through unsaturated fats, polyphenols, potassium, fiber and improved cardiometabolic health. Nevertheless, vascular improvement should not automatically be labeled direct neuroprotection. In many human studies, blood pressure, endothelial function or inflammatory biomarkers are intermediate outcomes, and their relationship to long-term neurological disease remains probabilistic rather than deterministic (2, 14).

### 2.3 The microbiota-gut-brain axis

The microbiota-gut-brain axis comprises immune, endocrine, neural and metabolic communication between intestinal microbes, the gut and the central nervous system. Microbial metabolites include short-chain fatty acids, indole derivatives, secondary bile acids and compounds generated from dietary polyphenols. These metabolites can influence epithelial cells, immune cells, enteroendocrine signaling and vagal pathways. The axis also operates in the opposite direction because stress, disease, medicines and autonomic activity alter gastrointestinal function and microbial ecology (7, 15).

Evidence linking microbial metabolites to microglial biology is especially influential. Erny et al. (8) showed that germ-free mice had immature and functionally altered microglia and that microbial short-chain fatty acids contributed to restoration of microglial features. Vailati-Riboni et al. (9) later reported that dietary inulin increased caecal short-chain fatty acids and partly counterbalanced age-related microglial dysregulation in mice. These studies support a mechanistic chain from fermentable substrate to microbial metabolite to microglial response. They do not, however, establish an effective dose for humans or prove that fecal short-chain fatty acid concentration reflects exposure of the brain. Related animal studies show that microbial metabolites can have divergent, context-dependent central effects. Erny et al. (8) demonstrated immature microglial profiles and impaired innate responses in germ-free mice, with partial restoration after complex recolonization or short-chain fatty acid exposure. Conversely, Sampson et al. (16) found that microbial short-chain fatty acids promoted microglial activation and motor abnormalities in an alpha-synuclein mouse model. Together, these studies reject the simplistic assumption that increasing every short-chain fatty acid is uniformly neuroprotective.

Polyphenols also participate in reciprocal microbiota interactions. A substantial proportion of ingested polyphenols reaches the colon, where microbial transformation generates smaller phenolic metabolites. The parent compound and its metabolites can alter microbial composition, while the microbiome influences systemic exposure and inter-individual response (1, 3).

Infection, microbial dysbiosis, impaired barrier integrity and oxidative stress can influence the central nervous system through interconnected immune, metabolic, neural and neurovascular pathways. As summarized in Table 1, these processes involve peripheral-to-central immune signaling, Toll-like receptor and inflammasome activation, reduced short-chain fatty acid production, microbial biotransformation of dietary compounds and blood-brain barrier dysfunction. Table 1 also distinguishes strongly supported mechanistic evidence from emerging or indirect human evidence, emphasizing that changes in microbial composition or intermediate biomarkers alone do not establish neurological causality (8, 11, 13).

**Table 1:** Mechanistic pathways linking infection, microbial dysbiosis and neuroinflammation in the central nervous system.

Initiating exposure or disturbance	Mechanistic pathway	Intermediate biological effect	Potential CNS implication	Evidence status	References
Systemic or neurotropic infection	Peripheral-to-central immune signaling	Cytokines, endothelial signals, vagal pathways, complement	Microglial and astrocytic activation; sickness behavior; synaptic stress	Strong mechanistic and animal evidence; outcome varies by pathogen and host	(10, 11)
Microbial products or barrier failure	TLR and inflammasome signaling	Lipopolysaccharide, TLR4, NF-kB, NLRP3, IL-1beta	Amplified inflammatory signaling and oxidative stress	LPS models represent inflammatory exposure, not complete live infection	(4, 64)
Low fermentable-fiber intake or dysbiosis	Reduced microbial metabolite production	Acetate, propionate, butyrate; epithelial tight junctions	Higher systemic inflammatory tone and altered microglial homeostasis	Compelling animal data; sparse direct human neurological mediation	(7–9)
Altered microbial ecology	Microbiota-gut-brain communication	SCFAs, indoles, bile acids, immune and vagal signals	Changes in barrier, neuroimmune and behavioral pathways	Composition alone does not establish function or causality	(15, 65)
Polyphenol-rich foods	Microbial biotransformation and host signaling	Phenolic metabolites, Nrf2, NF-kB, MAPK	Potential redox and inflammatory regulation	Bioavailability and responder variability limit prediction	(1, 3, 66)
Oxidative and vascular stress	Neurovascular and blood-brain barrier dysfunction	Reactive species, nitric oxide, tight-junction proteins	Reduced perfusion, greater inflammatory access, neuronal vulnerability	Human studies often use intermediate rather than clinical outcomes	(11, 13)

### 2.4 Inflammasomes, complement, and glial crosstalk

Inflammasomes provide a molecular bridge between infection-associated signals and sterile inflammatory stress. The NLRP3 inflammasome can be primed by signaling pathways that increase transcription of NLRP3 and pro-interleukin-1 beta, and then activated by a second set of cellular disturbances that may include potassium efflux, mitochondrial dysfunction, lysosomal damage and reactive oxygen species. Once assembled, the complex activates caspase-1 and promotes maturation of interleukin-1 beta and interleukin-18. This sequence is relevant to nutritional research because several phytochemicals influence upstream redox and kinase pathways in experimental systems. It is nevertheless important to distinguish inhibition of one measured inflammasome marker from a complete change in disease trajectory. Most dietary studies do not demonstrate target engagement in the human brain, and peripheral inflammasome measures cannot be assumed to represent microglial activity (4, 6).

The complement system is another example of an immune pathway with protective and potentially damaging functions. Complement components participate in pathogen recognition and clearance, while complement-tagged synapses can be removed by microglia during development and disease. In a prolonged inflammatory environment, excessive or mistimed complement activity may contribute to synaptic vulnerability. A dietary exposure that lowers a circulating inflammatory marker does not necessarily normalize complement-dependent synaptic processes. Consequently, complement is included in the framework as part of glial and immune crosstalk rather than as a dietary target for which clinical efficacy has been demonstrated (6, 12).

Astrocytes influence extracellular glutamate, ion balance, antioxidant support and metabolic coupling between blood vessels and neurons. Their responses to infection or systemic inflammation are shaped by signals from microglia, endothelial cells and infiltrating immune cells. Activated astrocytes can contribute to cytokine production and altered neurotransmitter handling, but they may also restrict injury and support repair. This functional diversity mirrors the limitations of the old binary description of microglia. Nutritional studies should therefore avoid interpreting a reduction in a single astrocytic marker as uniformly beneficial. A useful outcome panel would include inflammatory mediators, cellular metabolism, barrier measures and functional neurological endpoints (6, 17). Astrocyte-specific evidence also supports the need to distinguish cell type and metabolite. In experimental autoimmune encephalomyelitis, Rothhammer et al. (17) reported that antibiotic exposure worsened recovery-phase disease, whereas supplementation with microbiota-derived tryptophan metabolites reduced central inflammation through astrocytic aryl hydrocarbon receptor signaling. This model demonstrates a defined microbial-metabolite-astrocyte pathway, although it is neither a dietary trial nor an infection model.

Microglia, astrocytes and the neurovascular unit form a reciprocal network. Endothelial activation can increase leukocyte adhesion and cytokine transport; glial mediators can alter vascular permeability; and metabolic stress can amplify inflammatory signaling in all three compartments. Polyphenols, short-chain fatty acids and antioxidant nutrients may influence parts of this network, but their effects can differ by cell type and exposure concentration. The same compound may be anti-inflammatory in one model and inactive or even disruptive in another. This variability is not a reason to dismiss mechanistic research; it is a reason to report cell type, model, concentration, exposure time and measured pathway with precision (11, 13, 17).

The timing of intervention is also critical. Preventive exposure before an inflammatory challenge tests whether a compound changes susceptibility, whereas administration after the challenge tests modification of an established response. Many preclinical studies use pretreatment designs that are easier to control but less comparable with clinical care, where dietary habits precede illness and therapeutic decisions occur after symptoms begin. Studies should therefore distinguish long-term dietary conditioning, acute pretreatment and post-insult intervention. Combining these designs under the general label of neuroprotection exaggerates the consistency of the evidence (16, 17).

Taken together, inflammasome, complement and glial-crosstalk evidence supports a network model of neuroinflammation. It does not support the claim that one antioxidant or one anti-inflammatory food can switch off the process. Functional plant foods are more plausibly understood as repeated, low-intensity exposures that may alter metabolic and inflammatory thresholds within a broader dietary pattern. Whether those changes are large enough to affect clinical neurological outcomes remains an empirical question, especially during severe infection when pathogen factors, treatment timing, organ dysfunction and intensive-care exposures may dominate the biological response (4, 6, 12).

### 2.5 Systemic inflammation, metabolic stress, and neurological vulnerability

Systemic inflammation can affect neurological function without direct pathogen entry into the central nervous system. Cytokines and microbial products alter endothelial signaling, autonomic function, sleep, appetite and energy metabolism. Acute sickness behavior can be adaptive because it reallocates energy toward immune defense, but persistent inflammation may contribute to delirium, fatigue, mood disturbance or cognitive difficulty. These outcomes are heterogeneous and are influenced by age, frailty, vascular disease, prior neurological status and the severity of the systemic illness. Dietary research should therefore define the neurological phenotype rather than treating all post-infectious symptoms as one outcome (10, 11).

Metabolic health modifies this relationship. Insulin resistance, obesity, hypertension and dyslipidaemia can raise baseline inflammatory tone and impair endothelial function. A plant-rich dietary pattern may reduce neurological vulnerability indirectly by improving these upstream conditions. Such indirect mediation is clinically important, but it differs from direct suppression of microglial activation. Reviews should state whether an observed benefit is likely to arise through vascular-risk reduction, improved nutrient adequacy, microbial metabolism or a central cellular pathway. Failure to separate these routes creates an impression of mechanistic certainty that the available studies do not provide (2, 14).

Aging adds another layer because immune responses, barrier integrity, vascular function and the microbiota change over time. Older adults may have lower dietary diversity, impaired absorption, multimorbidity and greater medicine exposure. They may also be more vulnerable to delirium and prolonged functional decline after infection. The finding that dietary fiber partly counterbalanced age-related microglial changes in mice is therefore biologically relevant, but translation requires human studies that measure baseline diet, frailty, medicines and infection severity rather than age alone (9).

The background diet determines the meaning of a dietary intervention. Adding a polyphenol-rich food to a fiber-poor, energy-excess diet may produce a different response from replacing an ultra-processed snack with fruit, nuts or legumes. In the latter case, benefit may arise from both addition and displacement. Dietary trials should document the comparator, total energy intake and changes in other foods. Without this information, an apparent bioactive effect may be a substitution effect or an improvement in overall diet quality (18, 19). Human interventions support upstream modulation without proving central mediation. Wastyk et al. (19) randomized 36 healthy adults to high-fiber or high-fermented-food diets for 17 weeks. High fiber increased microbiome carbohydrate-active enzymes but did not change the prespecified cytokine-response score, whereas fermented foods increased microbial diversity and reduced inflammatory markers. The contrast shows that substrate delivery, baseline ecology and food processing influence immune response.

This systemic perspective narrows the claim appropriately. Plant foods may support a metabolic, vascular and microbial environment that is less permissive to excessive inflammation. That proposition is stronger than claims about treatment of a specific neurological infection because it aligns with the exposure and outcomes actually measured in most human studies. It also explains why infection-related neurological injury is treated later as an application of the framework rather than as the primary organizing category (18, 20).

## 3 Bioactive components and food-derived substrates

Plant-derived foods contain diverse bioactive compounds that may influence neuroinflammatory, oxidative, metabolic and microbiota-mediated pathways relevant to central nervous system health. As presented in Table 2, the principal classes include polyphenols, fermentable fiber, carotenoids, mushroom-derived compounds, terpenoids, stilbenes, organosulfur compounds and alkaloids, with proposed actions involving NF-κB, MAPK, Nrf2, inflammasome, microbial metabolite and redox-regulatory pathways. Table 2 also shows that, despite substantial experimental evidence, clinical translation remains limited by low bioavailability, variable metabolism, differences in food matrices, formulation-specific effects and the frequent non-equivalence of whole foods and concentrated supplements.

**Table 2:** Major plant-food bioactive classes, dietary sources, proposed mechanisms and translational limitations.

Bioactive class or food-derived substrate	Representative food sources	Principal proposed mechanisms	Human and experimental evidence	Major translational limitation	References
Polyphenols and flavonoids	Berries, citrus, tea, cocoa, coffee, legumes, herbs and spices	NF-kB/MAPK/Nrf2 and selected NLRP3-related signaling; microbial biotransformation	Extensive cell and animal evidence; mixed human cognitive and biomarker results	Dose, metabolism, food matrix and low exposure to parent compounds	(1, 4, 22)
Fermentable fiber and prebiotics	Legumes, oats, barley, fruits, vegetables, whole grains, resistant starch	SCFA production; epithelial and immune regulation; microglial homeostasis	Strong mechanistic animal evidence; growing human microbiome evidence	SCFA measurement and causal mediation remain difficult in humans	(7–9)
Carotenoids and antioxidant vitamins	Colorful fruits and vegetables, nuts, seeds and plant oils	Nutrient adequacy, redox enzymes, membrane protection	Dietary patterns are supportive; high-dose supplementation trials are inconsistent	Food effects cannot be inferred from isolated supplement trials	(30, 31)
Mushroom beta-glucans and ergothioneine	Edible and culinary-medicinal mushrooms	Innate immune-receptor signaling and redox-related effects	Predominantly compositional and preclinical evidence	Species, extraction, contamination and limited clinical trials	(34, 35)
Terpenoids and diarylheptanoids	Turmeric, rosemary, sage, citrus and other herbs or spices	Multiple inflammatory and redox targets	Promising preclinical evidence for curcumin and related compounds	Low bioavailability and formulation-specific exposure	(36, 37)
Stilbenes	Grapes, berries and peanuts	Sirtuin-associated, inflammatory and metabolic pathways	Mechanistic evidence exceeds consistent clinical evidence	Rapid metabolism and low parent-compound bioavailability	(38, 39)
Organosulfur compounds and alkaloids	Garlic, cruciferous vegetables, coffee, tea and black pepper	Redox, enzyme, neurotransmitter and absorption effects	Context-specific evidence; often evaluated as extracts	Dose, interactions and non-equivalence of foods and concentrates	(6, 12)

### 3.1 Polyphenols and flavonoids

Polyphenols comprise multiple structural classes, including flavonoids, phenolic acids, stilbenes and lignans. Food sources include berries, citrus fruits, grapes, tea, cocoa, coffee, legumes, whole grains, herbs and spices. Quercetin, catechins, anthocyanins, hesperetin, resveratrol and curcumin are frequently studied, although curcumin is chemically a diarylheptanoid rather than a flavonoid. Experimental studies report modulation of NF-kB, MAPK, Nrf2 and inflammasome pathways, reductions in selected cytokines and changes in antioxidant-enzyme activity (5, 21, 22).

NLRP3 is a useful focus because it links microbial products, mitochondrial stress, reactive species and interleukin-1 beta maturation. Hung et al. (4) reviewed evidence that dietary phytochemicals can interfere with priming or activation of the NLRP3 inflammasome in neurological models. The wording must remain selective: most evidence concerns isolated compounds in cells or animals, not ordinary consumption of the source food, and effects are not uniform across polyphenols. The claim supported by current evidence is that selected phytochemicals can modulate NLRP3-related signaling under experimental conditions, not that all polyphenol-rich foods suppress neuroinflammation in humans.

Flavonoids may also influence the microbiota and be converted to metabolites with different absorption and biological activity. This makes exposure difficult to infer from food composition tables alone. Urinary or plasma metabolites can improve exposure assessment, but many trials do not measure them. The clinical relevance of a parent compound may therefore be overestimated when *in vitro* experiments use the unmetabolised molecule at concentrations that are not sustained in circulation (1, 3, 23). Controlled human data illustrate both measurable effects and outcome specificity. In 90 older adults with mild cognitive impairment, Desideri et al. (24) compared approximately 990, 520 and 45 mg/day cocoa flavanols for 8 weeks. High- and intermediate-dose groups completed Trail Making Tests faster than the low-dose group, but Mini-Mental State Examination scores did not differ. Improvements in insulin resistance explained about 40% of composite cognitive-score variation, supporting vascular-metabolic mediation rather than demonstrated suppression of brain inflammation.

A comparative study of goji berry and ashwagandha extracts provides cellular evidence but is not a clinical trial. Zhang and Wang (25) reported increased proliferation of rat neural stem cells and reduced death in an MPP+-challenged neuronal cell model. The study supports hypotheses about cellular neuroprotection and extract combinations; it does not establish human efficacy, safety, dosage or brain exposure. Likewise, Yao's (26) network-pharmacology analysis linked goji constituents with aging-related targets, but computational target networks remain hypothesis-generating rather than physiological evidence.

### 3.2 Dietary fiber and prebiotics

Dietary fiber includes chemically and physiologically diverse carbohydrates that resist digestion in the small intestine. Fermentability, viscosity, particle structure and food matrix determine how a fiber affects transit, glycaemia, microbial ecology and metabolite production. Legumes, oats, barley, fruits, vegetables, whole grains, nuts and seeds provide mixtures of soluble and insoluble fibers. Resistant starch, inulin-type fructans and selected oligosaccharides are frequently used as prebiotic substrates (7, 19).

The most coherent neurological pathway is not a direct action of fiber on the brain but a sequence involving microbial fermentation, short-chain fatty acids, epithelial and immune responses, and downstream neuroimmune signaling. Butyrate can support colonic epithelial energy metabolism and tight-junction function and can inhibit histone deacetylases at sufficient concentrations. Acetate and propionate also participate in host metabolism and receptor signaling. The net effect depends on dose, substrate, microbial community, intestinal region and host condition, so the generic statement that more short-chain fatty acid is always better is not justified (7, 9). In a lead-exposure rodent model, dietary fiber attenuated microbiota disturbance and neuroinflammatory measures, but this model does not establish a general human effect (27).

Human evidence remains less direct than the animal evidence. Fecal short-chain fatty acids are influenced by production, absorption and transit, and higher fecal concentration does not necessarily mean greater host exposure. Intervention studies should therefore combine diet records with plasma or portal-relevant metabolites where feasible, microbial functional measures, barrier markers and neurological outcomes. The present evidence supports fiber as a plausible upstream modulator of neuroinflammatory vulnerability, not a proven treatment for neurological disease. Reviews of dietary microbiota interventions reach the same conclusion: mechanistic plausibility exceeds disease-specific human evidence (28). In the 82-participant, 8-week isocaloric trial by Meslier et al. (18), Mediterranean-diet assignment lowered plasma cholesterol and altered microbial carbohydrate-degradation pathways, but neurological outcomes were not measured. A 17-participant crossover pilot by Nagpal et al. (29) found that 6-week modified Mediterranean-ketogenic and American Heart Association diets produced different microbiome and fecal short-chain fatty acid profiles associated with cerebrospinal-fluid Alzheimer biomarkers. The small sample and correlational mediation preclude causal neurological conclusions.

### 3.3 Carotenoids, vitamins, and minerals

Plant foods supply vitamin C, folate, potassium, magnesium and carotenoids, while nuts and seeds provide vitamin E and minerals. These nutrients contribute to normal antioxidant defense, one-carbon metabolism, membrane stability and immune function. Deficiency correction is biologically important, but it should not be conflated with pharmacological supplementation above nutritional requirements. Observational associations between nutrient-rich diets and cognition may reflect the combined food pattern rather than an isolated antioxidant (30, 31).

Large supplementation studies and systematic reviews have not consistently reproduced the expected cognitive benefit of antioxidant hypotheses. Vitamin E did not delay progression from mild cognitive impairment to Alzheimer's disease in the trial by Petersen et al. (31), and long-term vitamin E, vitamin C and beta-carotene supplementation did not slow cognitive change among women at cardiovascular risk in the Women's Antioxidant Cardiovascular Study (30). These findings do not negate the value of antioxidant-rich foods, but they show that improving an oxidative-stress biomarker or providing a high-dose antioxidant is not sufficient evidence of neurological protection. Large trials provide important counterevidence to isolated-antioxidant claims. Petersen et al. (31) randomized 769 participants with amnestic mild cognitive impairment to vitamin E, donepezil or placebo for 3 years; vitamin E did not reduce progression to Alzheimer's disease (hazard ratio 1.02, 95% confidence interval 0.74–1.41). In 2,824 older women followed cognitively for 5.4 years, Kang et al. (30) found no slowing of cognitive change with vitamin E or beta-carotene, and vitamin C did not alter longitudinal change. These null findings strengthen the distinction between nutrient adequacy, whole-food patterns and pharmacological supplementation.

The distinction between food and supplement is therefore essential. Whole foods provide micronutrients together with fiber, water, unsaturated fats and phytochemicals, and they can replace less favorable foods. Supplements usually provide a narrow exposure, often at a dose unrelated to dietary intake. This difference helps explain why pattern-level dietary evidence can coexist with negative single-antioxidant trials (30–33).

### 3.4 Bioactive constituents of edible mushrooms

Edible mushrooms are fungi rather than plants, but they are retained because they are commonly included in plant-forward functional-food discussions. Their constituents include beta-glucans, ergothioneine, phenolic compounds, terpenoids, sterols and other polysaccharides. Beta-glucans can interact with innate immune receptors, while ergothioneine has redox-related properties. Culinary-medicinal mushroom research remains dominated by compositional studies, extracts and preclinical models (34, 35).

The appropriate conclusion is that mushroom constituents provide mechanistic candidates for immunomodulatory and neuroprotective research. Evidence is insufficient to rank edible mushrooms above other food groups for prevention of neurological disease, and concentrated mushroom products require quality control because species identification, cultivation, contamination and extraction influence composition. The section therefore avoids the earlier category of “mushroom neuroprotection” and instead discusses defined constituents and evidence boundaries (34, 35).

### 3.5 Terpenoids, alkaloids, and organosulfur compounds

Terpenoids occur in herbs, spices, citrus peels and edible plants. Curcumin from turmeric, carotenoids and multiple essential-oil constituents are often grouped within broad phytochemical discussions, although they differ substantially in chemistry and pharmacokinetics. Alkaloids such as caffeine and piperine affect neurotransmission, metabolism or absorption. Organosulfur compounds from garlic and cruciferous vegetables influence redox and inflammatory pathways. These classes should not be discussed as interchangeable antioxidants (36–38).

Curcumin illustrates the translation problem. It modulates multiple inflammatory targets in preclinical studies, including microglial pathways, but oral exposure to unformulated curcumin is limited by poor solubility, rapid metabolism and elimination. Azzini et al. (37) concluded that preclinical anti-neuroinflammatory evidence is promising while bioavailability and scarcity of robust human trials remain major obstacles. Novel formulations can increase measured exposure, but a formulation-specific result cannot be generalized to turmeric used as a culinary ingredient.

Resveratrol provides a parallel example. It is absorbed but extensively metabolized, producing low systemic availability of the parent compound (38). Human studies have not consistently replicated *in vitro* observations, partly because circulating metabolites, dose and tissue exposure differ from cell-culture conditions (39). These limitations should temper claims based on resveratrol-rich foods or supplements and reinforce the need to distinguish target engagement from clinical benefit. Formulation studies quantify why food and supplement evidence cannot be pooled. Shoba et al. (40) reported that 20 mg piperine given with 2 g curcumin increased short-term curcumin bioavailability by approximately 2,000% in healthy volunteers, demonstrating altered exposure rather than neurological efficacy. In a separate 42-person study, 1 g/day resveratrol for 4 weeks inhibited phenotypic indices of CYP3A4, CYP2D6 and CYP2C9 and induced CYP1A2 activity (41). Thus, exposure enhancement can simultaneously increase target engagement and interaction risk.

### 3.6 Whole-food matrices, culinary use, and combined exposures

A bioactive compound is consumed within a physical and chemical matrix that influences release, digestion, absorption and microbial transformation. The matrix of a berry, whole grain, legume, nut, spice or mushroom differs from that of a capsule or solvent extract. Cell walls can limit immediate release while delivering material to the colon; fats can modify absorption of lipophilic compounds; processing can increase or reduce accessibility; and cooking can change both nutrient content and palatability. These factors make food composition an incomplete proxy for biological exposure. A table that lists milligrams of a polyphenol without describing the food, preparation and dose can therefore imply a level of precision that is not present (1, 3).

Fruits and vegetables provide mixtures of fiber, vitamins, minerals and phytochemicals rather than isolated antioxidants. Berries are frequently studied because of their anthocyanins, citrus fruits because of flavanones, and leafy vegetables because of folate, carotenoids and other constituents. Yet the neurological interpretation should remain outcome-specific. A trial may measure memory, executive function, endothelial response or inflammatory markers, and improvement in one domain does not establish broad neuroprotection. Variation in cultivar, storage, processing and background diet further limits direct comparison across studies (22, 42, 43).

Nuts and seeds combine unsaturated fats, fiber, protein, vitamin E, minerals and polyphenols. Their vascular and cardiometabolic effects offer an indirect route to brain health, while their fiber and polyphenols may interact with the microbiota. The peanut trial by Parilli-Moser et al. (44) reported selected memory and stress-response findings in healthy adults, but one food-specific study cannot establish prevention of cognitive decline or infection-related neurological damage. Nut evidence is best interpreted within the wider pattern of repeated intake, energy balance and substitution for less favorable snacks (14, 45).

Legumes and whole grains provide fermentable substrates, resistant starch, plant protein and micronutrients simultaneously, so their effects are not captured by a narrow antioxidant model. Ye et al. (46) cataloged structurally diverse constituents from food-medicine homologous Leguminosae species; however, many entries concerned medicinal species or preparations that are not equivalent to ordinary portions of beans, peas or lentils. The chemical evidence therefore supports candidate mechanisms without implying that every legume food delivers a clinically active dose of each constituent.

Herbs and spices can increase phytochemical diversity, but culinary exposure is often much lower than doses used in experimental extracts. Turmeric supplies curcuminoids, black pepper supplies piperine, garlic supplies organosulfur precursors and tea or coffee supplies multiple phenolics and alkaloids. Combination can alter exposure; for example, piperine is frequently used to increase curcumin bioavailability, but an increase in exposure can also alter medicine interactions. Culinary use should therefore be separated from formulated supplement use in both evidence tables and safety statements (36, 37).

Fermented plant foods add another layer because microbial processing before consumption can alter metabolites, acidity and food structure. However, fermented does not automatically mean probiotic, and a product should not be described as microbiome-restorative without demonstrating viable organisms, functional metabolites or a reproducible host effect. Likewise, a prebiotic effect requires selective utilization by host microorganisms that confers a health benefit; it should not be inferred solely from the presence of fiber (19, 28).

Combined exposures may be additive, synergistic, antagonistic or simply independent. Fiber can support microbial communities that metabolize polyphenols, while polyphenols can influence microbial growth and enzyme activity. Unsaturated fats may change the absorption of lipophilic phytochemicals, and food processing may alter both. These interactions justify pattern-level research, but they also make it difficult to identify a single active component. Synergy should be treated as a hypothesis that requires factorial or mechanistic testing rather than as an assumed property of whole foods (3, 19).

The food-matrix perspective also helps reconcile apparently conflicting evidence. A population with high intake of minimally processed plant foods may benefit through improved nutrient adequacy, lower energy density, better vascular risk and greater microbial substrate diversity. A high-dose supplement containing one constituent may fail because it lacks these co-exposures, has poor absorption, reaches the wrong tissue or activates compensatory metabolism. Conversely, a formulated product may achieve pharmacological exposure that ordinary food cannot provide, which means its efficacy and safety must be assessed as a distinct intervention rather than used to validate a food claim (30, 32, 33). Taken together, these food-matrix, dose and metabolism constraints explain the evidence classifications consolidated in Table 2.

## 4 Convergent mechanisms of neuroinflammatory modulation

**Figure 1:**
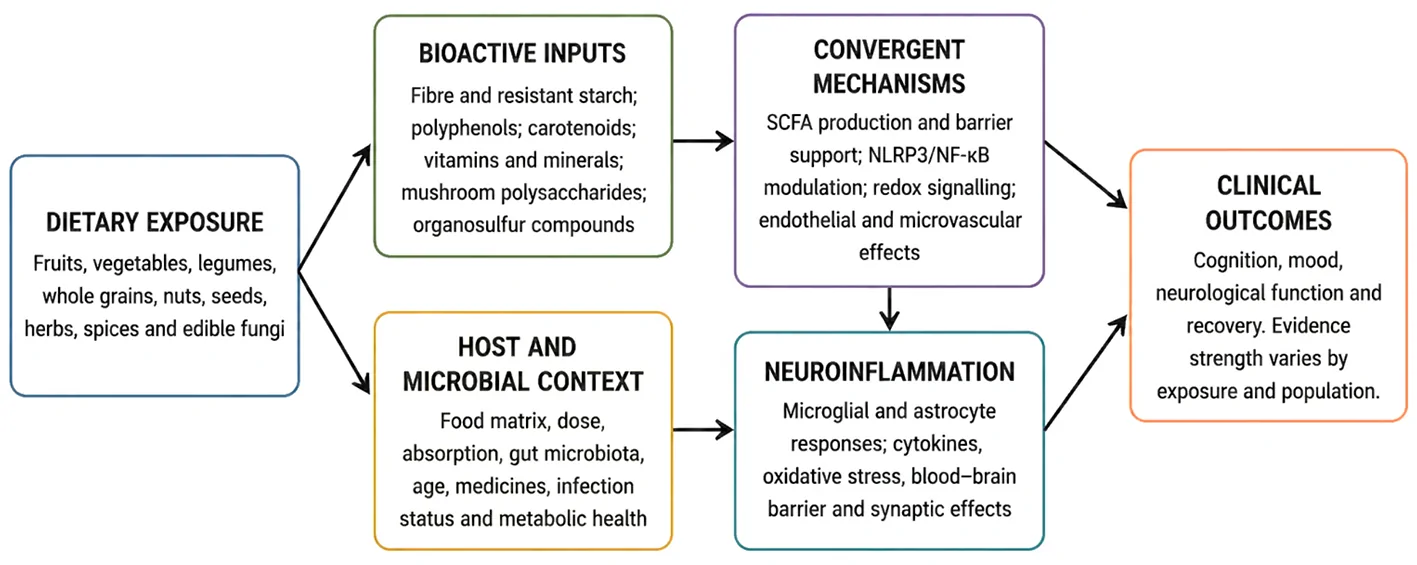
Conceptual framework linking functional plant foods, host context, neuroinflammatory mechanisms, and clinical outcomes.

Figure 1 illustrates how dietary exposure to functional plant foods provides bioactive compounds whose biological effects are modified by the food matrix, absorption, gut microbiota, age, medication use, infection status and metabolic health. These factors influence convergent inflammatory, oxidative, neurovascular and immune pathways that may affect neuroinflammation, cognition, mood, neurological function and recovery, although evidence for infection-specific neurological outcomes in humans remains limited (4, 7, 18).

### 4.1 Microglial NLRP3 and cytokine signaling

Activation of the NLRP3 inflammasome generally requires a priming signal that increases expression of inflammasome components and a second signal that promotes assembly and caspase-1 activation. Mature interleukin-1 beta and interleukin-18 can then amplify inflammatory responses. Mitochondrial dysfunction, ion flux, lysosomal stress and microbial products are among relevant triggers. Because these processes occur in immune cells inside and outside the brain, NLRP3 offers a mechanistic bridge between systemic inflammation, microbial signals and neuroinflammation (4).

Selected polyphenols reduce NLRP3-related markers in experimental models, but the evidence does not show selective suppression in all infection models or at ordinary dietary doses. A credible translational programme would measure dietary exposure, circulating metabolites, inflammasome activity in relevant cells and neurological outcomes within the same study. Without this chain, statements about NLRP3 remain mechanistic explanations rather than demonstrated mediators of dietary benefit (4, 6).

The goal of nutritional modulation should also not be described as complete immune suppression. Inflammasome activity contributes to antimicrobial defense and tissue responses. The desirable effect, if achievable, will be resolution of excessive or persistent signaling without impairing pathogen clearance. This balance is rarely assessed in studies that report only cytokine reduction after a phytochemical intervention (4, 11).

### 4.2 Fiber-derived short-chain fatty acids and barrier function

Fiber-derived short-chain fatty acids may influence neuroinflammatory vulnerability through several linked sites. In the colon, they contribute to epithelial metabolism and can regulate tight-junction and mucus-related processes. In immune cells, they signal through G-protein-coupled receptors and can affect gene expression. In animal models, microbial metabolites influence microglial maturation and inflammatory responsiveness. Reduced translocation of microbial products may also lessen peripheral signals reaching the brain (7, 8). This pathway is attractive because it connects a realistic food exposure with a microbial product and host response. Nevertheless, it is not yet justified to call SCFAs the established main mechanism of cognitive protection during systemic inflammation in humans. Fiber modifies glycaemia, satiety, bowel function, bile-acid metabolism and food displacement in addition to microbial fermentation (19, 29). The strongest experimental fiber-to-microglia evidence remains model-specific. Vailati-Riboni et al. (9) fed adult and aged mice diets containing 1% cellulose or 2.5% or 5% inulin for 8 weeks. Inulin increased caecal short-chain fatty acids and largely restored age-associated microglial gene expression and tumor necrosis factor-alpha secretion toward adult profiles. However, Wastyk et al. (19) observed no average improvement in the primary cytokine-response score after a high-fiber intervention in humans, showing that animal target engagement cannot be assumed clinically.

### 4.3 Oxidative stress and cellular defense

Oxidative stress is generated when reactive species exceed cellular control and repair. During infection or chronic inflammation, immune cells deliberately produce reactive species, while mitochondrial dysfunction can sustain additional production. Neurons are vulnerable because of high energy demand, long cellular processes and lipid-rich membranes. Plant-food constituents may influence redox balance through Nrf2-regulated enzymes, metal chelation, membrane effects and indirect improvement of metabolic health (42, 43).

Direct radical scavenging is an incomplete explanation because many phytochemicals circulate at low concentrations and are rapidly metabolized. The more plausible interpretation for several compounds is modulation of endogenous stress-response pathways. Even then, antioxidant effects in cells do not predict cognitive benefit. Negative or inconsistent supplementation trials show that oxidative stress is not a single target that can be corrected reliably with one high-dose nutrient (30, 31).

The review of *Phyllanthus emblica* by Wu et al. (47) identifies polyphenols, tannins and related antioxidant mechanisms, but it synthesizes oxidative-stress and aging models rather than demonstrating infection-specific neuroprotection. It is therefore used as supportive mechanistic literature, with the same translational qualifications applied to other phytochemical reviews.

### 4.4 Neurovascular and metabolic mediation

Plant-rich diets can improve cardiometabolic exposures that are themselves relevant to brain health, including blood pressure, lipid profile, insulin sensitivity and endothelial function. Nuts, seeds, legumes, whole grains and unsaturated plant oils contribute to this pattern. Some apparent neuroprotective effects may therefore be mediated by vascular risk reduction rather than direct action of a phytochemical on neural tissue (2, 14, 45).

This indirect route is clinically important rather than a weakness. However, mechanistic precision requires authors to state whether an intervention changed cerebral perfusion, systemic vascular function, inflammation or cognition. Combining these outcomes under the general label neuroprotection obscures the level at which evidence was obtained. Future trials should pre-specify mediators and use causal analysis where sample size permits (18, 24). Human trials support this indirect mediation more consistently than direct neuroimmune target engagement. Meslier et al. (18) observed lower cholesterol and increased insulin sensitivity alongside diet-related microbiome changes, while Desideri et al. (24) linked cognitive-test differences partly to improved insulin resistance. Neither study measured microglial activation, so their results support a cardiometabolic route to neurological resilience rather than direct central anti-inflammatory efficacy.

### 4.5 Metabolic, epigenetic, and mitochondrial interfaces

Neuroinflammatory responses depend on cellular metabolism. Activated immune cells alter glucose use, mitochondrial function and lipid handling to support cytokine production, phagocytosis and repair. Persistent metabolic disturbance can increase reactive species and maintain inflammatory signaling. Food-derived metabolites may influence this interface through receptors, redox-sensitive transcription factors and enzyme inhibition. The evidence is strongest for defined experimental pathways and weakest for claims that a dietary pattern produces a specific epigenetic state in the human brain (7, 38).

Butyrate is often described as a histone-deacetylase inhibitor. This mechanism is biologically plausible and is demonstrated in multiple experimental contexts, but the concentration at a target tissue matters. Most colonic butyrate is used by colonocytes or metabolized before reaching systemic circulation. It is therefore inappropriate to assume that a fiber intervention produces brain concentrations comparable with those used in cell culture. Human studies should measure relevant metabolites and downstream effects rather than relying on a mechanistic label alone (7).

Polyphenols and their metabolites can influence Nrf2-related antioxidant responses, NF-kB signaling, mitochondrial enzymes and apoptosis pathways. The direction and magnitude of effect vary by compound and metabolite. Because metabolites may differ structurally from the parent compound, a cell experiment using quercetin, resveratrol or curcumin does not necessarily model the dominant circulating exposure after food consumption. Pharmacokinetic sampling is consequently part of mechanism testing, not an optional addition (36, 38, 39).

### 4.6 Integrated interpretation of convergent pathways

The pathways described above are connected rather than parallel. A fiber-rich food can change microbial metabolism, epithelial integrity, circulating inflammatory signals and vascular function. A polyphenol-rich food can contribute fermentable material, undergo microbial conversion and influence host signaling. Improvement in cardiometabolic health can reduce inflammatory pressure on the neurovascular unit. The same dietary pattern can therefore act at several points without any one pathway accounting for the full outcome (18–20). The resulting conclusion is deliberately conditional. Functional plant foods can modulate processes relevant to neuroinflammation under experimental and some human conditions. The probability of a clinically meaningful outcome depends on exposure, baseline risk, duration, adherence, microbiome function, vascular status and the outcome selected (19, 32, 33). Figure 1 therefore represents a testable causal chain, not a claim that every arrow has been demonstrated in humans. The most decisive future studies should measure the food exposure, circulating or microbial metabolite, intermediate inflammatory or vascular change and neurological endpoint in the same participants (7, 18, 19).

## 5 Evidence from preclinical, clinical, and epidemiological studies

**Figure 2:**
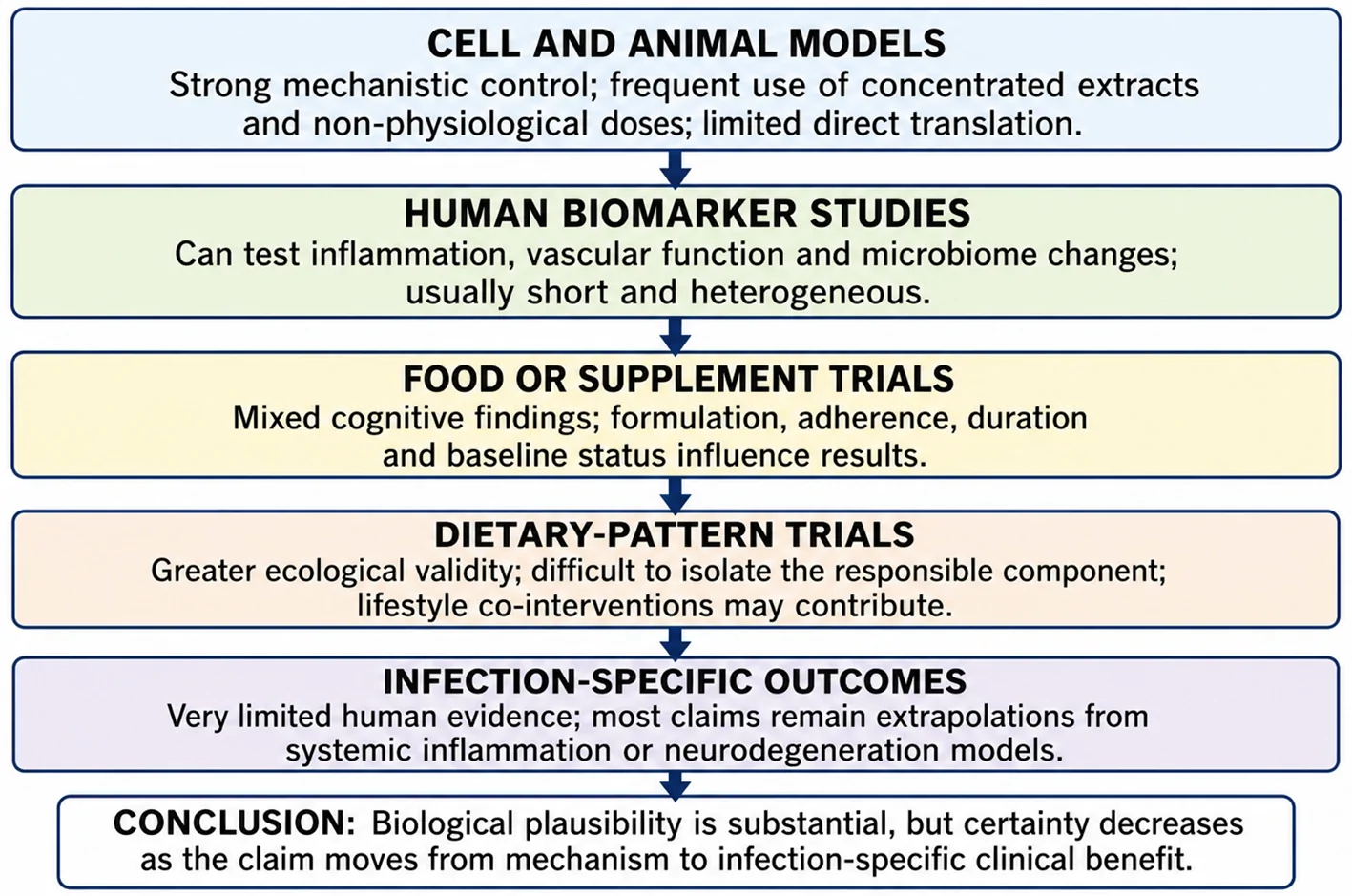
Evidence chain for functional plant foods and neuroinflammation.

Figure 2 shows the progression of evidence from controlled cell and animal studies through human biomarker studies, food or supplement trials, and dietary-pattern trials to infection-specific neurological outcomes. Although mechanistic plausibility is substantial, certainty decreases along the evidence chain because human studies are heterogeneous and direct evidence for infection-specific clinical benefits remains very limited (8, 13, 32).

### 5.1 Strengths and limits of preclinical evidence

Cell and animal studies are indispensable for identifying targets, testing temporal relationships and sampling tissues that are difficult to study in humans. They provide evidence that polyphenols can alter inflammatory signaling, that microbiota-derived metabolites affect microglia, and that dietary fiber can change microbial and immune phenotypes. They also permit controlled inflammatory challenges and comparison of genetically modified models (4, 8, 9).

Their limitations are equally important. Extract concentrations may exceed dietary exposure, compounds may be delivered by injection or gavage, and purified diets can create effects that differ from ordinary food patterns. Rodent microbiota and metabolism do not replicate human variation. Behavioral tests are proxies rather than direct measures of human cognition or daily function. An LPS challenge reproduces aspects of innate immune activation but not pathogen replication, tissue tropism, adaptive immunity, antimicrobial therapy or clinical complications. Preclinical results should therefore be described as mechanism-supporting rather than clinically confirmatory (13, 16, 17).

The cellular study of goji berry and ashwagandha extracts illustrates both the value and limitation of controlled models. Rat neural stem cells and an MPP+ neuronal model permit direct assessment of proliferation and survival, but the extracts are complex, absorbed constituents are uncertain and the outcomes are not equivalent to prevention of Parkinson's disease. The study is therefore treated as mechanistic evidence rather than evidence for dietary treatment (25). The Leguminosae review by Ye et al. (46) similarly catalogs 25 constituents from food-medicine homologous species and summarizes proposed effects on oxidative stress, inflammation, apoptosis, mitochondria and cerebral blood flow. Its value is taxonomic and mechanistic. Because it is a review of heterogeneous constituents and models, It is not treated as evidence that consumption of all legumes produces those effects or that medicinal preparations are equivalent to commonly eaten pulses.

### 5.2 Human dietary-pattern evidence

Human evidence is most persuasive when dietary exposure is sustained, adherence is measured and outcomes are clinically relevant. In a PREDIMED cognitive substudy, Mediterranean diets supplemented with extra-virgin olive oil or nuts were associated with better cognitive outcomes than a control diet among older adults at high cardiovascular risk (33). The trial supports a dietary-pattern effect but does not identify whether polyphenols, unsaturated fats, vascular improvement, social contact or other components were responsible. The sample was also specific and the cognitive substudy had limitations related to baseline testing and attrition. Among 447 randomized participants, 334 completed follow-up after a median 4.1 years; the Mediterranean-plus-olive-oil group improved frontal and global composites relative to controls, while the nut-supplemented group improved memory, but attrition differed between groups (33).

The 3-year MIND diet trial provides an important counterweight to optimistic observational findings. Barnes et al. (32) found that cognition improved in both intervention and control groups, with no significant between-group difference in the primary cognitive outcome. Both groups received guidance and reduced energy intake, which may have narrowed contrast. The result does not prove that dietary quality is irrelevant; it shows that a plausible pattern may fail to produce an incremental benefit under a particular trial design and population. In the 604-participant MIND trial, 3-year global cognition increased by 0.205 standardized units in the intervention group and 0.170 units in controls; the adjusted between-group difference was 0.035 (95% confidence interval −0.022 to 0.092; *p* = 0.23) (32).

Microbiome-focused human interventions add intermediate evidence. In the NU-AGE study, a 1-year Mediterranean diet intervention in older adults changed microbiome patterns and was associated with reduced frailty and improved health indicators (20). Such studies support diet-microbiome interaction but remain unable to show that a specific microbial change caused a neurological outcome. Functional measurements, replication and mediation analysis are needed. Ghosh et al. (20) profiled 612 non-frail or pre-frail adults across five European countries over 12 months. Diet-responsive taxa were associated with lower frailty, better cognition and lower C-reactive protein and interleukin-17, but the analysis linked correlated changes and did not demonstrate that microbiome alteration mediated cognitive benefit.

Smaller food trials can reveal specific effects but should not be overgeneralized. Parilli-Moser et al. (44) reported improvements in aspects of memory and stress response after peanut or peanut-butter consumption in healthy young adults, with associations involving polyphenol intake and fecal short-chain fatty acids. The trial was small, involved a young healthy population and used several outcomes; it therefore provides a signal rather than definitive evidence of disease prevention. The ARISTOTLE trial randomized 63 healthy adults to 25 g/day roasted peanuts, 32 g/day peanut butter or a phenolic-free control butter for 6 months. Immediate memory improved in both peanut groups, but multiple outcomes, small groups and a healthy sample limit generalizability (44).

### 5.3 Observational evidence and residual confounding

Prospective cohorts often associate Mediterranean, MIND-like or plant-forward patterns with slower cognitive decline or lower dementia risk. These studies can assess habitual intake over long periods, but diet is measured with error and healthy behaviors cluster. Education and income influence food access, healthcare, cognitive testing and survival. Physical activity, smoking, sleep and cardiometabolic risk may be incompletely measured. Statistical adjustment reduces but cannot eliminate residual confounding (48). Observational effect sizes can be substantial yet remain non-randomized. Morris et al. (48) followed 923 adults for an average of 4.5 years; compared with the lowest MIND-adherence tertile, the middle and highest tertiles had adjusted Alzheimer's-disease hazard ratios of 0.65 (95% confidence interval 0.44–0.98) and 0.47 (0.26–0.76). These associations justify trials but cannot exclude socioeconomic, behavioral or reverse-causation explanations.

Reverse causation is also possible. Early cognitive or neurological changes can alter shopping, cooking, appetite and food preference before diagnosis. Excluding early cases and repeating dietary assessment can reduce this problem, but few studies can remove it completely. Conclusions should therefore distinguish association from intervention effects and avoid implying that the observed dietary pattern is the sole causal factor (32, 48).

### 5.4 Supplements, bioavailability, and formulation

Curcumin and resveratrol show why preclinical potency can fail to translate. Oral curcumin is poorly soluble and extensively metabolized, while resveratrol is rapidly conjugated after absorption. Formulations with lipids, nanoparticles, phospholipids or absorption enhancers may increase exposure, but each formulation becomes a distinct intervention with its own safety and pharmacokinetics. Findings cannot be transferred automatically to the parent food or to another supplement (37, 38).

Clinical trials of polyphenol supplements vary in dose, duration, cognitive status and outcome. Selective positive findings coexist with null results, and publication bias may favor small studies with multiple outcomes. Reviews should report this heterogeneity rather than using the number of mechanistic papers as a proxy for efficacy. Ayaz et al. (36) emphasized pharmacokinetic challenges and limited clinical translation for several lead phytochemicals despite extensive preclinical evidence. Antioxidant supplementation illustrates a broader lesson. A biological mechanism can be valid while an intervention based on that mechanism fails because timing, tissue exposure, disease stage, target selection or compensatory biology is wrong. The appropriate response is not to dismiss oxidative or inflammatory pathways, but to avoid claiming that antioxidant-rich foods and high-dose antioxidant pills are clinically equivalent (30, 31).

### 5.5 Overall strength of evidence

The evidence is strongest for the following propositions: plant-rich diets deliver fermentable substrates and diverse bioactives; diet modifies the gut microbiome and cardiometabolic environment; microbial short-chain fatty acids regulate microglial features in animal models; selected phytochemicals alter inflammatory pathways in experimental systems; and some dietary-pattern trials improve intermediate or cognitive outcomes. Evidence is moderate and heterogeneous for sustained cognitive protection from specific plant-food patterns. Evidence is weak for assigning benefit to a single bioactive within a whole diet and very weak for prevention or treatment of infection-specific neurological complications in humans (8, 20, 32, 33). This graded conclusion is more informative than a general statement that plant foods are good for brain health. It identifies where evidence is actionable, where it is hypothesis-generating and where the review must remain agnostic. It also avoids treating the absence of direct infection-specific evidence as a reason to repeat disclaimers throughout every subsection. The boundary is stated once at the conceptual and clinical levels and then applied consistently (32, 33).

### 5.6 Formulated polyphenols in human trials

Human trials of curcumin illustrate formulation-specific and outcome-specific signals. A 4-week trial of a solid-lipid curcumin preparation reported acute or chronic improvements in selected attention, working-memory and mood measures in healthy older adults, while an 18-month trial of bioavailable curcumin in non-demented adults reported improvements in selected memory and attention outcomes and exploratory positron-emission tomography findings (49, 50). These studies are important because they demonstrate that measurable human effects are possible, but both tested proprietary formulations rather than culinary turmeric, involved modest samples and do not establish prevention or treatment of a neurological disease. Cox et al. (49) randomized 60 adults aged 60–85 years to 400 mg solid-lipid curcumin or placebo and reported selected acute and 4-week effects. Small et al. (50) randomized 40 non-demented adults to 90 mg curcumin twice daily or placebo for 18 months; verbal long-term retrieval improved within the curcumin group, while several between-group results were borderline or exploratory. Neither trial established disease modification.

Resveratrol trials are similarly mixed. Witte et al. (51) reported improved memory performance and hippocampal functional connectivity in healthy older adults after 26 weeks, whereas a 52-week phase II trial in mild-to-moderate Alzheimer's disease found that high-dose resveratrol was generally tolerated and altered selected biomarker trajectories without demonstrating a clear clinical disease-modifying effect (52). The appropriate synthesis is not that resveratrol succeeds or fails universally, but that population, formulation, exposure, comparator and outcome determine interpretation. These human data reinforce the need to avoid transferring supplement findings to the source food. Witte et al. (51) studied 46 overweight older adults for 26 weeks and observed improved word retention (*p* = 0.038) with 200 mg/day resveratrol. By contrast, Turner et al. (52) randomized 119 patients with Alzheimer's disease for 52 weeks, escalating resveratrol to 1,000 mg twice daily; biomarker changes and brain-volume loss occurred without demonstrated clinical disease modification, and nausea, diarrhea and weight loss were common adverse events.

Outcome hierarchy remains essential. Peripheral inflammatory markers and vascular measures can support systemic target engagement; cognitive tests provide greater neurological relevance but are sensitive to practice effects and multiplicity; and clinically meaningful change in diagnosis, disability or daily function provides the strongest evidence. Safety, adverse events and withdrawal also belong in the efficacy assessment, particularly for older adults with polypharmacy (32, 52, 53).

### 5.7 Why preclinical promise and human trials diverge

The first source of divergence is dose. Experimental studies often use purified compounds at concentrations selected to produce target effects. Ordinary foods may deliver much lower systemic exposure, and metabolism can transform the compound before it reaches the tissue. A negative food trial does not necessarily invalidate the cellular mechanism, but it may show that the mechanism is not engaged at the tested exposure. Conversely, a high-dose supplement cannot be used to infer the effect of the source food (38, 40, 52).

The second source is formulation. Curcumin has low and variable oral bioavailability in many conventional preparations, while resveratrol is absorbed but extensively metabolized, producing low exposure to the unchanged parent compound. Formulations designed to increase exposure can differ substantially in excipients and pharmacokinetics. Trials that group all curcumin or resveratrol products together obscure these differences. Reviews should report formulation, dose and metabolite evidence whenever these compounds support a conclusion (37–39).

The third source is model mismatch. A toxin-induced or genetically engineered animal model isolates selected features of a human condition. Lipopolysaccharide induces innate immune activation but does not reproduce pathogen replication, antimicrobial treatment, organ failure or adaptive immunity. Behavioral tests in rodents do not map directly onto human cognitive syndromes. Preclinical evidence is therefore strongest for mechanism and weaker for prediction of treatment efficacy (10, 16, 17).

The fourth source is heterogeneity among people. Age, genetics, baseline diet, microbiome function, nutrient status, comorbidity, medicines and adherence alter response. An intervention may help participants with low baseline intake but add little when the comparator group also improves its diet. Precision-nutrition hypotheses are reasonable, but subgroup claims require prespecification and adequate power rather than *post hoc* division of a small trial (19, 32).

The fifth source is outcome timing. Cytokines and vascular markers can change within weeks, whereas prevention of cognitive decline may require years. A trial can be long enough for biomarker change but too short for clinical neurological separation. Attrition, practice effects and competing illness become more important as follow-up lengthens. Study duration should therefore be chosen from the causal model rather than convenience (32, 33, 49, 50).

The sixth source is publication and interpretation bias. Positive secondary outcomes and favorable preclinical studies are more likely to be highlighted than null findings. Antioxidant supplementation illustrates the danger of moving from a plausible mechanism to broad clinical expectations. The negative or inconsistent vitamin E evidence shows that oxidative stress is biologically relevant without proving that high-dose antioxidant supplementation modifies cognitive disease (30, 31).

Across these trials, positive findings are concentrated in selected tests, intermediate biomarkers or specific formulations, whereas larger or longer comparisons often narrow or nullify effects. This pattern supports cautious, outcome-specific interpretation rather than a pooled claim of neuroprotection (32, 52).

## 6 Infection-related neurological injury: a translational application

**Figure 3:**
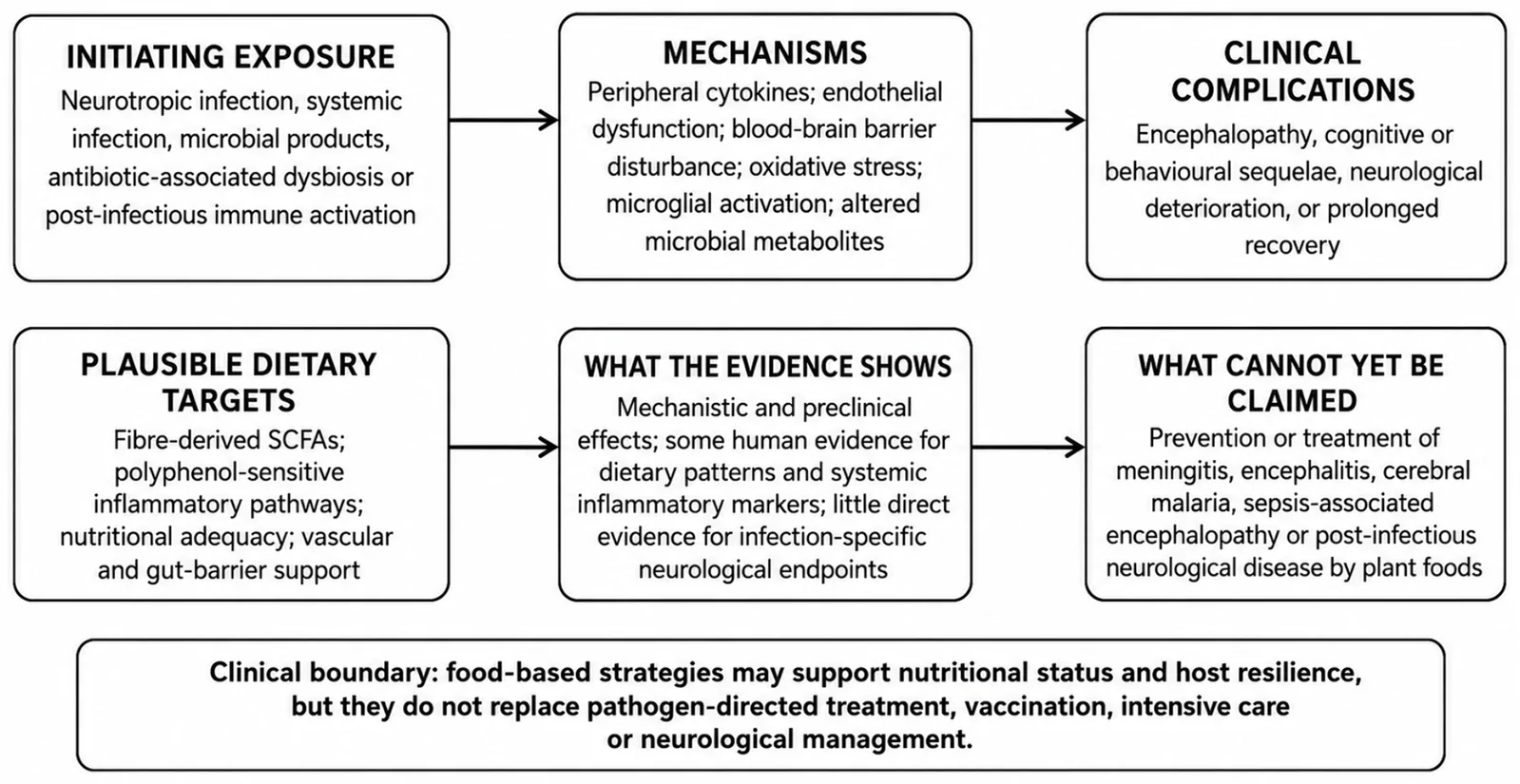
Infection-related neurological injury as an application of the general neuroinflammatory framework.

Figure 3 illustrates how neurotropic or systemic infections, microbial products, dysbiosis and post-infectious immune activation may produce neurological complications through inflammatory, vascular, oxidative, blood–brain barrier and microglial mechanisms. Although plant-food components may support nutritional adequacy, gut-barrier function and host resilience, current evidence does not establish that they prevent or treat infection-specific neurological diseases or replace vaccination, pathogen-directed therapy, intensive care or neurological management (11, 53).

### 6.1 Direct and indirect pathways

Infection-related neurological complications arise through multiple routes. Neurotropic pathogens may infect neural or meningeal cells directly. Systemic infection can produce encephalopathy through cytokines, endothelial dysfunction, altered perfusion, coagulation, metabolic disturbance, medicines and organ failure without direct invasion. Post-infectious syndromes may involve persistent immune activation or autoimmunity. These mechanisms differ clinically and should not be collapsed into a single outcome category (11, 54).

Plant-food bioactives are relevant to some shared mechanisms, particularly oxidative stress, endothelial function, gut-barrier integrity and inflammatory signaling. This relevance is indirect. For example, reducing an LPS-induced cytokine in a rodent brain supports an anti-inflammatory mechanism but does not show that a food changes mortality, seizures, hearing loss, cognitive sequelae or functional recovery after bacterial meningitis. The manuscript therefore avoids pathogen-by-pathogen sections that describe disease biology without corresponding dietary evidence (11, 54).

### 6.2 What infection models can and cannot show

Lipopolysaccharide models are widely used because TLR4 activation produces reproducible systemic and central inflammatory responses. They are useful for testing the priming of microglia, cytokine production and sickness behavior. However, live bacterial infection also involves replication, tissue invasion, antimicrobial defense and evolving pathogen burden. Viral and parasitic infections engage different receptors, cell types and vascular processes. Dietary effects observed after LPS challenge should therefore be labeled endotoxin-model findings rather than infection-treatment findings (10, 11).

The distinction also applies to antimicrobial assays. A plant extract can inhibit microbial growth *in vitro* at a concentration that is not achievable in blood, cerebrospinal fluid or tissue. Antimicrobial activity in a laboratory assay does not show prevention of neurological invasion or compatibility with standard drugs. Human infection studies must prioritize established antimicrobial therapy, supportive care and vaccination, while nutritional interventions are evaluated as adjunctive strategies with prespecified safety criteria (11, 36).

### 6.3 Current human evidence gap

Direct human evidence is sparse for plant foods and infection-specific neurological endpoints. Studies commonly measure infection incidence, general inflammation, nutritional status or recovery rather than neurological complications. Patients with meningitis, encephalitis, cerebral malaria or sepsis-associated encephalopathy are medically heterogeneous and often critically ill, making diet randomization difficult. Outcomes are also influenced strongly by pathogen, treatment delay, organ dysfunction and baseline health (11, 54).

The appropriate clinical hypothesis is therefore narrower: a nutritionally adequate, plant-rich background diet may reduce chronic inflammatory and vascular vulnerability and may support recovery, but it has not been shown to prevent or treat the acute neurological consequences of a defined infection. Trials could test adjunctive food-based interventions after clinical stabilization, focusing on feasibility, gut function, inflammatory resolution, cognition and functional recovery. Claims about antimicrobial substitution are excluded. The evidential boundary is therefore explicit: infection-related neurological injury is a bounded application of a broader neuroinflammatory framework, not the organizing framework for largely general or preclinical dietary evidence (11, 53).

### 6.4 Clinical contexts and the limits of extrapolation

Bacterial meningitis illustrates the distinction between mechanism and treatment. The neurological outcome is shaped by pathogen burden, antimicrobial timing, host inflammatory responses, intracranial complications and supportive care. Polyphenol activity in a microglial or endotoxin model cannot establish prevention of hearing loss, cognitive sequelae or mortality after meningitis. At most, it identifies pathways that might be considered in carefully designed adjunctive research after pathogen control and safety have been addressed. The present review therefore does not assign any plant food a disease-modifying role in bacterial meningitis (11).

Viral encephalitis and other neurotropic viral conditions are similarly heterogeneous. Some viruses directly infect neural or glial cells, while others produce vascular or immune-mediated complications. A food-derived compound may show antiviral or anti-inflammatory activity *in vitro* yet fail because achievable human exposure is insufficient, because it does not reach the infected compartment, or because suppression of inflammation interferes with viral control. Direct pathogen assays, pharmacokinetics and neurological outcomes are all necessary before a dietary constituent can be described as clinically relevant (54).

Sepsis-associated encephalopathy is often discussed as an example of indirect neurological injury during systemic infection. Its clinical expression can include delirium, altered consciousness and later cognitive or functional problems, but these outcomes arise within a complex state involving circulatory, metabolic, inflammatory, sedative and organ-failure effects. Long-term dietary quality may influence baseline vascular and metabolic resilience, yet there is no basis for claiming that a functional food treats acute sepsis-associated encephalopathy. Nutritional care in critical illness must prioritize established energy, protein, swallowing and organ-function considerations (53). Clinical outcome data demonstrate why infection-related neurological recovery cannot be reduced to a cytokine pathway. Pandharipande et al. (53) followed 821 adults treated for respiratory failure or shock; delirium occurred in 74%, and at 3 months 40% had global cognition scores comparable with moderate traumatic brain injury and 26% with mild Alzheimer's disease. Longer delirium duration independently predicted worse cognition at 3 and 12 months. Although not a diet study, these data define clinically meaningful endpoints for adjunctive nutrition trials.

Cerebral malaria involves parasite-related vascular sequestration, endothelial activation, inflammation and brain injury. Antioxidant or endothelial mechanisms may appear relevant, but mechanistic overlap is not evidence of clinical benefit. A dietary intervention would need to be evaluated alongside prompt antimalarial treatment, supportive care and age-specific risks. The absence of direct controlled human evidence is especially important in severe infections where delay or substitution of effective treatment can be fatal (55).

Post-infectious cognitive or fatigue syndromes present a different research opportunity because diet can be studied during recovery rather than during uncontrolled pathogen replication. Even here, case definition, time since infection, baseline health and concurrent rehabilitation must be specified. A plant-forward diet could plausibly improve nutrient adequacy, cardiometabolic health or gastrointestinal symptoms, but improvement should not automatically be attributed to reduction of central neuroinflammation. Trials should combine patient-centered function with exposure biomarkers and mechanistic measure (53).

Antibiotic-associated disruption of the microbiota is another plausible application. Antibiotics can alter microbial composition and metabolite production, but the direction and duration of change depend on the drug, dose, infection, age and previous microbiome. Fiber-rich foods may support recovery of microbial function, yet immediate tolerance can be limited during gastrointestinal illness, and indiscriminate use of supplements may be inappropriate. Studies should define the antibiotic exposure and measure function rather than promising restoration of an ideal microbiome (56). Human antibiotic-perturbation data also show incomplete and individualized recovery. Dethlefsen and Relman (56) followed three adults for 10 months through two ciprofloxacin courses, analyzing more than 1.7 million bacterial sequences from 52 to 56 samples per person. Community composition changed rapidly, recovery differed between participants, and repeated exposure produced persistent effects in some taxa. Therefore, claims of universal microbiome restoration after fiber or fermented foods are premature.

Vaccination and pathogen prevention remain outside the therapeutic claims of this review but are important to the clinical hierarchy. A healthy diet supports general nutritional status, whereas vaccination, sanitation, vector control and antimicrobial stewardship directly prevent or control specific infectious threats. Presenting plant foods as alternatives would confuse supportive health behavior with pathogen-specific prevention. The revised figures place dietary support downstream of the evidence boundary rather than beside established infection control (11, 54).

Across these contexts, the same rule applies: the closer the outcome is to a named infection, the more direct the evidence must be. A reduction in lipopolysaccharide-induced interleukin-1 beta is mechanistic evidence. A change in systemic C-reactive protein is a human biomarker outcome. Prevention of encephalopathy, disability or death is a clinical endpoint. These levels should not be collapsed. The review retains infection because it is scientifically important, while concluding explicitly that infection-specific human efficacy is currently unproven (10, 11). Figure 3 should therefore be read from left to right as an evidential filter: shared pathways create hypotheses, but infection-specific prevention or treatment requires direct trials alongside standard antimicrobial and supportive care.

## 7 Sustainable dietary patterns, food-system translation, and equity

**Figure 4:**
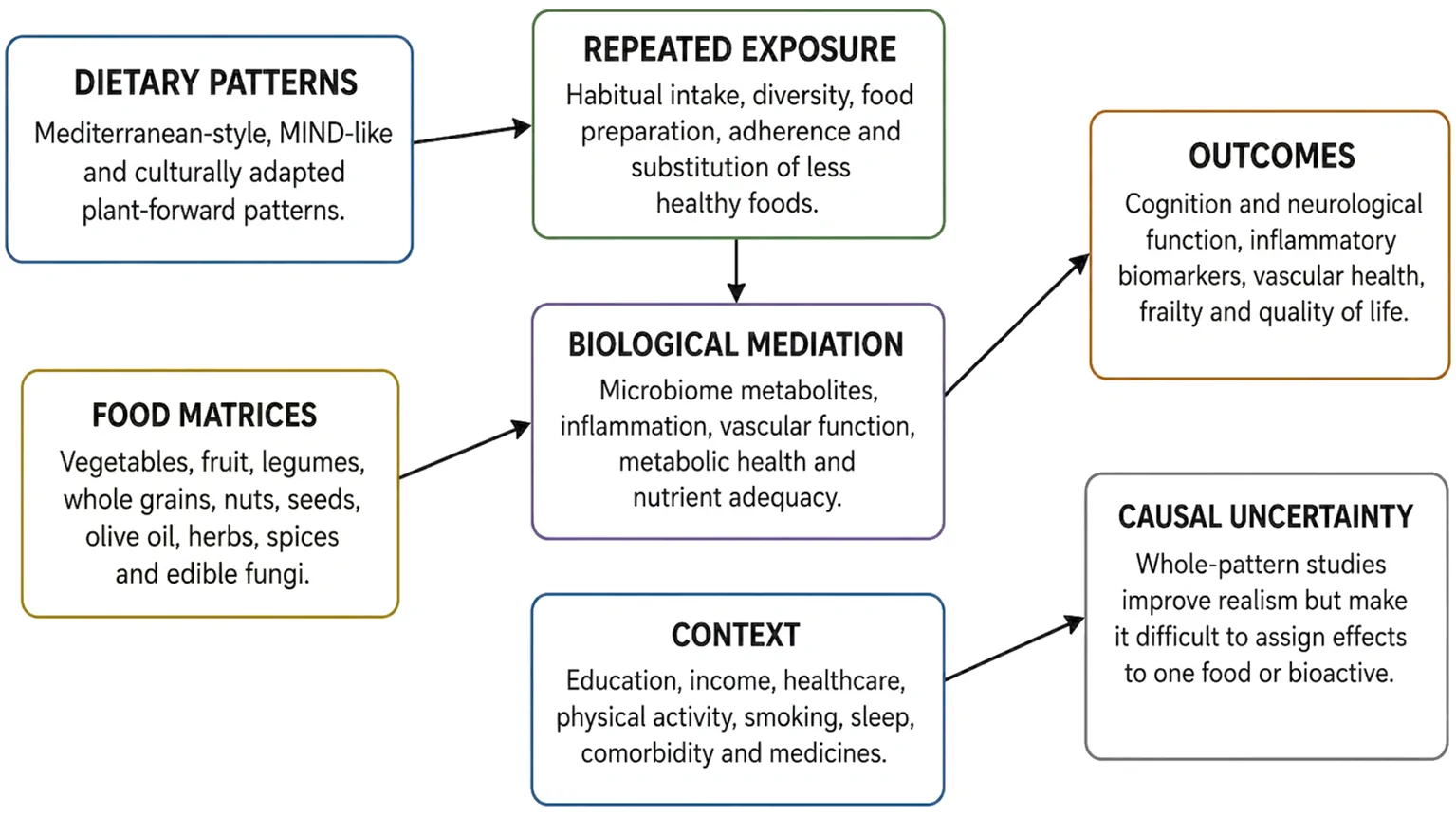
Integration of functional plant foods within whole dietary patterns, sustainable food-system translation, and the main sources of causal uncertainty.

Figure 4 shows how plant-forward dietary patterns and diverse food matrices may influence cognition, neurological function, inflammation, vascular health, frailty and quality of life through repeated exposure and biological mediation involving the gut microbiome, metabolic health and nutrient adequacy. However, these outcomes are also shaped by socioeconomic, behavioral and clinical factors, while whole-diet studies make it difficult to attribute observed effects to a single food or bioactive compound (18, 20, 33).

Whole dietary patterns are the most realistic exposure for public health because foods are consumed in combinations and because adding one food usually displaces another. Mediterranean-style patterns emphasize vegetables, fruits, legumes, whole grains, nuts, seeds and unsaturated oils, while MIND-like patterns prioritize selected foods associated with cognitive health. Their value lies in repeated exposure across microbial, vascular, metabolic and inflammatory pathways rather than in a single bioactive. Comparable principles can be implemented with locally available foods instead of being copied as a fixed regional menu (2, 57). This pattern-level framing is also emphasized in recent Mediterranean-diet syntheses (58).

Pattern-level interpretation has two advantages. Modest effects across multiple pathways may accumulate over time, and the intervention can improve nutrient adequacy, cardiometabolic risk and microbial substrate supply simultaneously. It also has an important limitation: a positive pattern trial cannot identify a single responsible constituent. Conversely, a null trial may reflect an active comparator, small dietary contrast, insufficient duration or already adequate baseline diet rather than biological irrelevance (18, 20, 32, 33). The PREDIMED and MIND trials illustrate the need for balanced interpretation. PREDIMED provides randomized evidence compatible with cognitive benefit from Mediterranean-style interventions, whereas the MIND trial found no significant incremental benefit in its primary cognitive outcome (32, 33). Differences in population, comparator, adherence, exposure contrast and attrition can yield different results. Both trials are more informative than a general statement that plant-rich diets protect the brain. The contrast between Morris et al. (48) and Barnes et al. (32) is especially instructive. A strong prospective association was not reproduced as a significant incremental 3-year trial effect when both groups received counseling, caloric restriction and weight-loss support. This divergence supports the manuscript's interpretation that baseline diet, comparator activity and behavioral co-interventions can determine apparent efficacy.

Culturally adapted plant-forward patterns should preserve nutritional and mechanistic principles rather than a list of imported foods. Legumes, whole grains, leafy vegetables, fruits, seeds, nuts where affordable, herbs and spices can provide fiber and phytochemical diversity in many settings. Food safety, affordability, preparation burden, seasonality and sustained adherence are more important than marketing a small number of superfoods (20, 59).

Legumes are especially relevant to both nutrition and food-system translation because they provide fiber, protein and minerals, can be stored with comparatively low post-harvest loss when handled well, and are embedded in many traditional cuisines. Ye et al. (46) illustrate the chemical diversity of food-medicine homologous Leguminosae species, but common pulses should not be equated with concentrated medicinal preparations. Their most defensible population role is as affordable dietary staples rather than as sources of presumed therapeutic doses.

A food-pattern strategy avoids the false choice between mechanism and practicality. Pragmatic trials can deliver culturally tailored diets while measuring adherence, polyphenol metabolites, microbial function, short-chain fatty acids, inflammatory markers, vascular outcomes, cognition, cost and food-source characteristics. Such designs are more informative than uncontrolled food-frequency associations or high-dose single-compound studies because they connect biological response with real-world feasibility (18–20). Across these pattern studies, the most credible synthesis is that diet can alter multiple upstream exposures, but benefit depends on the contrast actually achieved and on whether the comparator also improves. Table- and figure-level claims should therefore identify the measured outcome rather than label every favorable biomarker as neurological protection (18, 32, 33).

### 7.1 Environmental sustainability, affordability, and access

A plant-forward neurological-nutrition pattern cannot be described as sustainable solely because it contains plant foods. Environmental consequences depend on the food category, production system, yield, land-use change, water and nutrient management, energy use, processing, packaging, cold storage, transport and waste. At the level of broad food groups, greater reliance on legumes, whole grains, vegetables, fruits and nuts and lower reliance on resource-intensive foods can align health and environmental objectives; however, no individual plant product is automatically low impact (60–62). Environmental data also require distributional rather than categorical interpretation. Poore and Nemecek (63) synthesized information from approximately 38,700 farms and 1,600 processors, packaging operations and retailers across five environmental indicators. Impacts varied markedly among producers, although even lower-impact animal products generally exceeded plant substitutes. These findings support plant-forward substitution while rejecting the claim that every plant food or production system is uniformly sustainable.

Affordability and access affect both intervention success and observational interpretation. Hirvonen et al. (59) showed that the EAT-Lancet reference diet was unaffordable for many low-income populations, demonstrating that nutritional desirability does not ensure economic accessibility. People with greater income and education also have greater access to diverse foods, healthcare, safe environments and health information, which can contribute to observed diet-cognition associations. Trials may provide foods and counseling that are unavailable after the study; implementation research should therefore report cost, preparation time, market availability, seasonal variation and sustained adherence. Hirvonen et al. (59) used prices for 744 foods in 159 countries and estimated a global median cost of US$2.84 per day in 2011 purchasing-power-parity terms; the benchmark diet exceeded per-capita household income for at least 1.58 billion people. Affordability is therefore an intervention mechanism, not merely an implementation detail.

Sustainable translation also requires attention to substitution and supply chains. Replacing an energy-dense ultra-processed snack with a local fruit, pulse or nut may create nutritional and environmental benefits that are not captured by studying the added food alone. Conversely, importing a highly processed extract, refrigerated specialty beverage or scarce superfood may increase cost, packaging and energy demand while providing uncertain neurological value. Priority should be given to nutritionally adequate, safe and culturally acceptable foods whose production and distribution can be maintained without shifting environmental or economic burdens to other communities (60, 63).

Food safety is inseparable from sustainable neurological nutrition. Contamination, poor storage, adulteration and botanical misidentification can reverse expected benefits and generate avoidable waste. Mushrooms require reliable species identification; grains and nuts require moisture, mycotoxin and allergen control; and herbal products need identity and purity assurance. Sustainable recommendations therefore include safe production, appropriate processing, storage and quality control rather than assuming that traditional or natural origin guarantees safety (34).

Communication should avoid the language of superfoods. Emphasizing an imported berry, proprietary extract or supplement can increase inequity and distract from affordable staples that provide fiber, protein and nutrient diversity. Public-health guidance is stronger when it describes food groups, substitutions, preparation and waste reduction. This food-first framing also reduces the risk of converting a sustainable-diet discussion into marketing for concentrated products with uncertain efficacy (59, 63).

Equity should be built into research design. Populations with high infectious burden, food insecurity or limited neurological services are underrepresented in diet-brain trials, yet their baseline diet, microbiome, environmental exposures and treatment access may differ substantially from those of well-resourced cohorts. Studies should involve local investigators, use culturally valid neurological measures, evaluate locally available foods and report who bears the cost and labor of dietary change. A sustainable intervention must be biologically credible, affordable, safe, acceptable and feasible within the food system in which it will be used (59).

## 8 Safety, dose, and practical considerations

**Figure 5:**
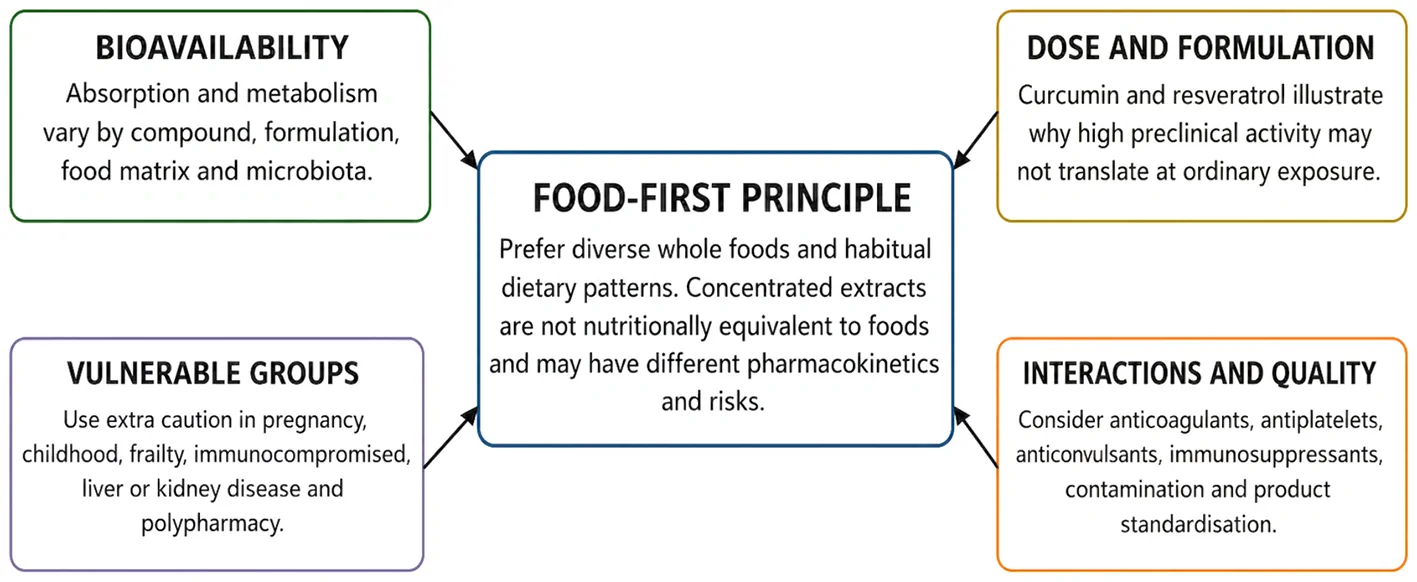
Food-first safety framework for functional plant foods and concentrated bioactives.

Figure 5 emphasizes a food-first approach in which diverse whole foods and habitual dietary patterns are preferred because the bioavailability and effects of plant bioactives vary according to the compound, formulation, food matrix and gut microbiota. Concentrated extracts require caution because appropriate doses remain uncertain, product quality and medicine interactions may affect safety, and vulnerable groups may face greater risks (40, 41, 52).

For ordinary foods, the principal recommendation is diversity, moderation and attention to nutritional adequacy. No universal neuroprotective dose has been established for berries, tea, cocoa, turmeric, mushrooms, nuts, seeds or other functional foods. Studies use different foods, extracts and outcome measures. Translating a milligram-per-kilogram animal dose to a culinary recommendation is inappropriate (32, 33, 44, 49).

Concentrated supplements require a separate safety assessment. Polyphenol products can interact with anticoagulants, antiplatelets, anticonvulsants, immunosuppressants and medicines metabolized by relevant enzymes or transporters. Piperine can alter drug exposure. Mushroom and herbal products vary in species, extraction and contamination. Patients with liver or kidney disease, pregnancy, childhood, frailty, immunocompromise or polypharmacy should not assume that a natural product is automatically safe (40, 41).

Bioavailability is not simply a technical obstacle to be overcome. A formulation that increases systemic exposure may also change toxicity or interactions. Curcumin and resveratrol products should therefore be reported by formulation, dose and pharmacokinetic evidence rather than by compound name alone (37, 38). The interaction evidence is quantitative, although not neurological. Shoba et al. (40) demonstrated a large, short-lived rise in curcumin exposure when piperine was co-administered, while Chow et al. (41) showed that pharmacological resveratrol altered several cytochrome P450 phenotypic indices in 42 volunteers. These studies support product-specific medication review whenever bioavailability enhancers or gram-dose polyphenols are used.

During active neurological infection, food-based support must follow clinical priorities. Swallowing safety, gastrointestinal function, energy and protein needs, renal or hepatic status, drug administration and infection-control requirements may determine what is appropriate. A general plant-rich diet is not a substitute for individual dietetic assessment in critical illness (53).

The practical conclusion is food first and claims last. Diverse minimally processed plant foods can be recommended within established dietary guidance because of their overall nutritional value. Disease-specific claims, high-dose extracts and combination products require direct evidence, quality control and clinical supervision (32, 33). Accordingly, Figure 5 separates ordinary food use from concentrated exposure, and the safety branch should be applied before any efficacy claim is transferred across formulations.

### 8.1 Product quality, regulation, and clinical communication

The regulatory category of an intervention affects the certainty with which composition and exposure can be interpreted. A whole food sold for ordinary consumption is not equivalent to a concentrated botanical extract, a standardized supplement or an investigational product. Labels may report the mass of an ingredient without identifying the active constituent, extraction solvent, degradation products or batch variability. For clinically relevant interpretation, studies should state botanical identity, plant part, extraction method, standardization marker, independent quality testing and storage conditions. Without these details, apparently similar products may deliver materially different exposures (37, 40, 41). Reporting should also distinguish nominal ingredient mass from measured parent compound and metabolites, because exposure can differ substantially even when labels use the same botanical name (40, 41).

Contamination and adulteration are especially important when a product is marketed for vulnerable patients. Potential concerns include heavy metals, pesticides, microbial contamination, undeclared pharmaceuticals and substitution of botanical species. The existence of a plausible neuroprotective mechanism does not offset poor product quality. Research reports should document certificates of analysis or equivalent quality procedures, and reviews should not treat unverified commercial preparations as interchangeable with controlled study materials (34).

Clinical communication should distinguish general dietary guidance from a disease-specific recommendation. Advising a varied diet rich in minimally processed plant foods is supported by broad nutritional and cardiometabolic evidence. Advising a patient to take a concentrated extract for neuroinflammation is a different claim that requires product-specific efficacy, safety and interaction data. This distinction is particularly important when patients are receiving anticoagulants, antiplatelets, anticonvulsants, immunosuppressants or multiple medicines (32, 33, 41).

Risk communication should also avoid implying certainty from the word natural. Natural products can produce pharmacological effects, and pharmacological effects can include adverse events. Conversely, the absence of proven disease-specific efficacy does not mean that the source food lacks nutritional value. Clear communication separates food quality, nutritional adequacy, mechanistic plausibility and therapeutic evidence so that one category is not used to imply another (40, 41, 52).

## 9 Discussion and research priorities

The evidence supports a narrower and more useful interpretation than a general claim that plant foods are beneficial for the brain. Fermentable fiber and selected phytochemicals can engage microbial, inflammatory, redox and vascular pathways relevant to neuroinflammation, but certainty decreases as the outcome moves from a molecular marker to human cognition, neurological disease or infection-specific complications. Whole dietary patterns have greater ecological validity than isolated compounds, whereas isolated-compound studies provide greater exposure specificity. Neither design alone resolves causality; progress depends on linking food intake to absorbed or microbially generated metabolites, target engagement and meaningful outcomes while preserving null findings.

Future research should begin with a defined causal question. Trials designed to test fiber-derived short-chain fatty acids should specify fiber type, food matrix, dose and duration, measure exposure and microbial function, and include barrier, inflammatory and neurological outcomes. Trials designed to test polyphenol-NLRP3 mechanisms should measure relevant metabolites and target engagement rather than assuming exposure from the assigned food.

Infection-related research requires a staged approach. First, preclinical studies should compare endotoxin models with live-pathogen models and evaluate pathogen burden, immune defense and neurological injury together. Second, observational clinical studies should identify whether habitual diet or microbiome function predicts recovery after controlling for infection severity and treatment. Third, carefully selected adjunctive interventions can be tested after stabilization, with safety and functional outcomes prioritized.

Human studies should be adequately powered and long enough for the proposed outcome. Cognitive decline generally requires longer follow-up than inflammatory biomarkers. Multiple testing should be controlled, outcomes registered in advance and null findings reported. Trials should include diverse populations because microbiome composition, diet, infection burden, food access and comorbidity vary across regions.

Multi-omics can improve mechanism but also increase false discovery. Microbiome sequencing should be combined with functional measures such as metabolites and pathways, not interpreted solely from taxonomic abundance. Metabolomics can establish whether a participant was exposed to a bioactive or its microbial products. Neuroimaging and fluid biomarkers can add specificity when justified, but clinically meaningful function remains essential.

Recent studies provide complementary but different evidence types. Zhang and Wang (25) report a cell-based extract comparison; Ye et al. (46) catalog constituents from Leguminosae species; Yao (26) maps potential goji targets computationally; and Wu et al. (47) synthesize oxidative-stress mechanisms for *Phyllanthus emblica*. Their contribution is mechanistic or hypothesis-generating, and none provides direct human evidence for infection-specific neurological benefit.

Reporting standards should identify whether the intervention is a food, extract, purified compound or formulated supplement. Botanical identity, processing, storage, dose, compliance and adverse events should be documented. Diet trials should describe the comparator and the foods displaced, because substitution may explain part of the effect. Data sharing and harmonized outcomes would improve replication and future meta-analysis.

### 9.1 Minimum design features for decisive studies

A decisive trial should begin with an explicit estimand: the effect of a defined food, compound or dietary pattern on a prespecified outcome in a specified population over a stated period. The comparator must be credible and described in equal detail. In a pattern trial, investigators should report both foods added and foods displaced. In a compound trial, they should report formulation, dose, pharmacokinetics and adherence. These features determine what causal claim can be made from the result.

Mechanistic mediation requires temporal ordering. The dietary exposure should first produce a measurable change in a metabolite or target pathway, followed by a change in an intermediate inflammatory, vascular or barrier measure and then a neurological outcome. Cross-sectional correlations among diet, microbiota and cognition cannot establish this sequence. Repeated sampling and prespecified mediation analysis can strengthen inference, although mediation remains sensitive to unmeasured confounding.

In infection-related studies, pathogen and treatment variables must be incorporated rather than treated as background noise. Relevant factors include pathogen type, diagnostic certainty, antimicrobial timing, severity, organ dysfunction, intensive-care exposures and rehabilitation. A nutritional association that disappears after adjustment for severity should not be promoted as neuroprotective. Conversely, a well-controlled signal during recovery could justify a subsequent adjunctive trial.

Finally, evidence synthesis should preserve negative findings and separate certainty by outcome. A review may conclude that evidence is strong for a cellular pathway, moderate for a systemic biomarker, limited for cognition and absent for an infection-specific neurological endpoint. This graded conclusion is more useful than a single statement that functional foods are beneficial. It also creates a transparent research agenda in which each new study addresses a defined missing link.

## 10 Conclusion

Plant-food components are relevant to neuroinflammation because defined exposures converge on microbial, inflammatory, redox and vascular pathways, not because the general value of plant-rich diets is novel. The most coherent mechanisms are modulation of microglial inflammatory signaling by selected phytochemicals and production of short-chain fatty acids from fermentable fiber. Experimental support is strong, while direct human mediation evidence and clinically meaningful effect estimates remain limited. Human evidence is mixed and should remain visible in the conclusion. Dietary-pattern and food trials report favorable, null and context-dependent cognitive, vascular, microbiome and inflammatory outcomes. Curcumin and resveratrol studies show that formulation and exposure can produce selective human signals without establishing universal efficacy or equivalence to the source food. Observational associations remain vulnerable to confounding and reverse causation. Infection-related neurological injury is a plausible but unproven application of the general framework. Infection is an initiating cause, neuroinflammation is one mechanism and neurological complications are outcomes. Plant foods cannot be claimed to prevent or treat named neurological infections without direct evidence. For Frontiers in Sustainable Food Systems, the most defensible contribution is a food-first framework that links neurological nutrition with affordability, cultural adaptation, product safety, environmental context and substitution within whole diets. Disease-specific and sustainability claims should both be proportional to the evidence.
